# PanaxGDB: A Comprehensive Platform for *Panax*

**DOI:** 10.3389/fpls.2022.883818

**Published:** 2022-05-25

**Authors:** Yuan Lin, Bing Hao, Ying Chun Lu, Yang Dong, Ying Li, Guang Hui Zhang, Zi Jiang Yang, Gui Sheng Xiang, Guan Ze Liu, Xue Jiao Li, Qin Zhu, Qing Hui Yang, Xu Zhen Li, Sheng Chao Yang

**Affiliations:** ^1^State Key Laboratory of Conservation and Utilization of Bio-Resources in Yunnan, The Key Laboratory of Medicinal Plant Biology of Yunnan Province, National & Local Joint Engineering Research Center on Germplasms Innovation & Utilization of Chinese Medicinal Materials in Southwest China, Yunnan Agricultural University, Kunming, China; ^2^Key Laboratory of Medicinal Plant Biology, Yunnan Agricultural University, Kunming, China; ^3^College of Agronomy and Biotechnology, Yunnan Agricultural University, Kunming, China; ^4^College of Biological Big Data, Yunnan Agriculture University, Kunming, China

**Keywords:** *Panax*, database, cultivars, genome, saponins

## Abstract

The genus *Panax* is a valuable natural medicinal source used worldwide that contains high levels of triterpenoid saponins with extensive pharmacological activities. In past decades, molecular biotechnology and breeding techniques have been respectively used to generate omics data and information on cultivars primarily from *Panax ginseng* (ginseng), *Panax quinquefolium* (American ginseng), and *Panax notoginseng* (Sanqi) to biosynthesize valuable saponins, improve product quality, and conduct cost-controlled cultivation. Although much data have been produced, there are concerns that redundant data might be generated and that relatively scattered data might be overlooked. Therefore, many scientists desire a reliable, comprehensive omics database of the *Panax* genus that could save time and promote integrated analysis. Therefore, to provide all-inclusive, reliable, and valuable information on the *Panax* genus, PanaxGDB, an open comprehensive database that integrates data on omics and information on varieties, was established. The database contains information on nearly 600 compounds from 12 *Panax* species, draft genomic sequences with annotations and gene expression levels, single nucleotide polymorphisms, genome-wide association analysis based on agronomic traits, globally collected germplasm information, summaries, omics data of the *Panax* genus, and online versatile analytic tools. The *Panax* genus database will be updated when new data are released to continue serving as a central portal to boost research on the biology and functions of *Panax*. PanaxGDB is available at: http://panaxGDB.ynau.edu.cn.

## Introduction

The genus *Panax* (Araliaceae) consists of 16 species primarily distributed in southwestern and northeastern Asia and North America. The herb has been used as a traditional medicine for treating cardiovascular and cerebrovascular diseases and improving immune systems for thousands of years (Zuo et al., 2017). Three high-value species, *Panax quinquefolius, Panax ginseng*, and *Panax notoginseng*, have been successfully domesticated and are widely cultivated in Asia and North America to meet increasing market demands (Beedata, 2020; Zhang et al., 2020; Ibaogao, 2021). In 2019, the total global market for *P. notoginseng, P. ginseng*, and *P. quinquefolius* exceeded $9.34 billion (Beedata, 2020; Ibaogao, 2021). With the market increasing tremendously, several excellent cultivars are cultivated to fulfill high-quality requirements (Zhang et al., 2020). Evaluation standards and certification platforms used to select plants for breeding vary in different regions and countries, resulting in poor exchange of cultivars information and popularity of cultivars, which slows research on breeding.

Saponins are responsible for various pharmacological activities in the *Panax* genus (Xu et al., 2019). In 12 species of *Panax*, 492 damarane-type and oleanane-type saponins have been identified. Damarane-type saponins are classified based on different carbon skeletons as protopanaxadiol-type, protopanaxatriol-type, and ocotillo-type. Different chemical configurations and ligand modifications of those saponins generate many differences in physiological and pharmacological functions (Chen et al., 2009; Nag et al., 2012). There are large differences in the types of saponins among different cultivars and species of *Panax*. Damarane-type saponins are found in most species of *Panax*, except *Panax stipuleanatus*, and ocotillo-type saponins have not been detected in *P. notoginseng* (Zhu et al., 2004). Oleanane-type saponins are primarily distributed in *P. stipuleanatus, Panax japonicus, Panax major*, and *Panax zingiberensis* (Yang et al., 2014). Other compounds with pharmacological activity include dencichine for hemostasis and flavonoids for antibacterial, antidiabetic, and anti-inflammatory effects (Ding et al., 2018; Chen et al., 2019; Liu et al., 2020; Wang et al., 2020b).

Although market demand for saponins is increasing, supplies are unable to meet the demand because of the long planting period of *Panax*. Therefore, there have been attempts to biosynthesize saponins using yeast in the past years, which requires large amounts of omics information on saponin biosynthesis (Wang et al., 2020a). Morphological differences among the related species are slight, and as a result, it is difficult to identify species such as *Panax bipinnatifidus* and its wild relatives (Zhou et al., 2020). To date, there are seven versions of genome assemblies and abundant RNA-seq data for *Panax* species, which have been used to uncover mechanisms underlying the biosynthesis of ginsenosides or to identify genes associated with agronomic traits (Jayakodi et al., 2018; Yang et al., 2021). The large omics data sets have been used to identify several UDP-glycosyltransferases involved in the saponin biosynthesis pathway in *P. ginseng, P. notoginseng*, and *P. zingiberensis* (Wang et al., 2015, 2016; Tang et al., 2019; Jiang et al., 2021). In addition, 67 chloroplast genomes of *Panax* species and cultivars were identified in phylogenetic studies (Kim et al., 2015a,b). However, further research is still needed on the relations among *Panax* species and phylogenetic relations resulting from complex population differentiation. Although much of the *Panax* omics data have been reported in dispersive publications and databases, the information utilization is inefficient.

**Figure 1:**
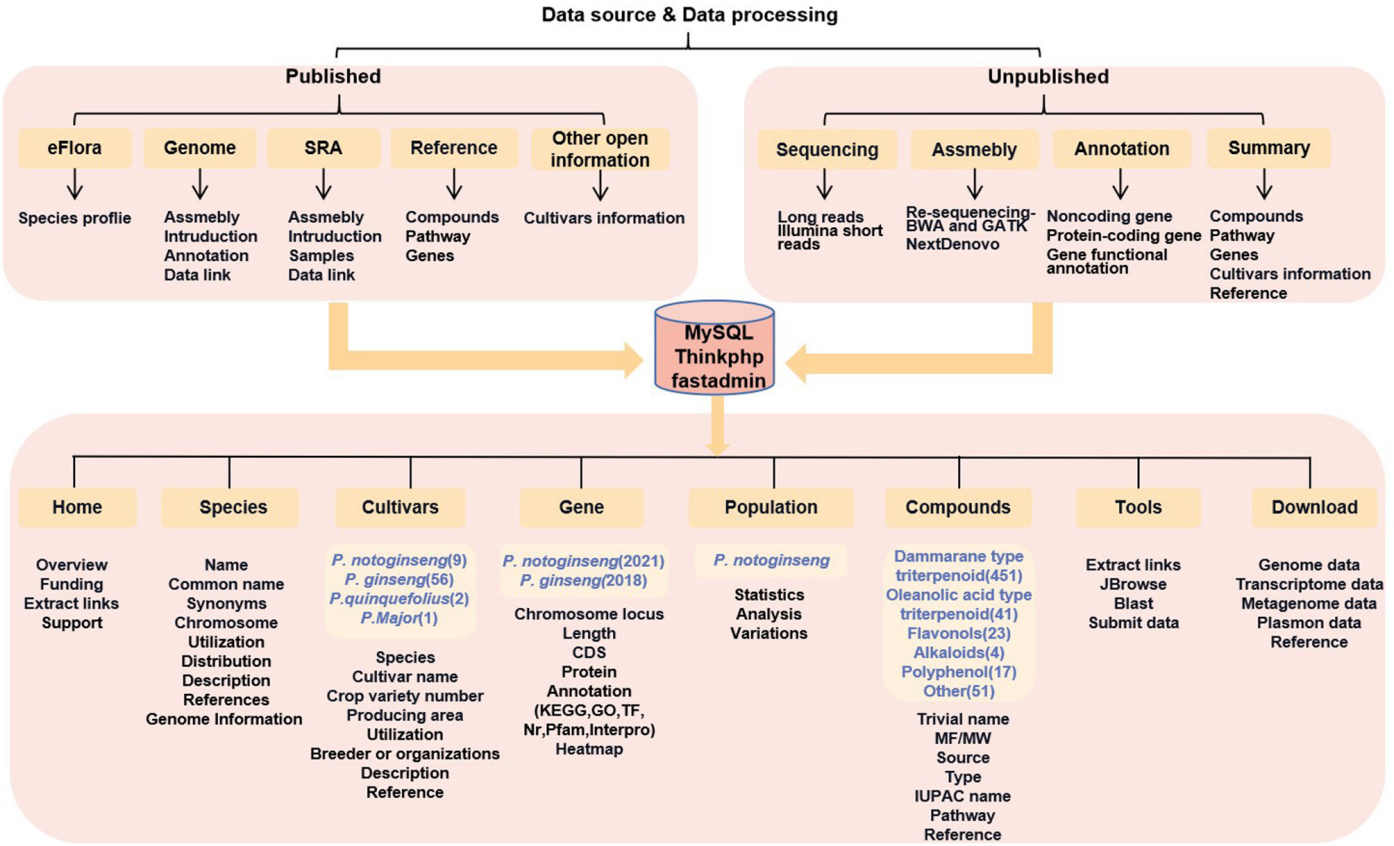
A schematic diagram of the architecture of the PanaxGDB.

A *Panax* comprehensive database is needed to provide better support and for research to progress more effectively on cultivation techniques and mechanisms affecting the quality of pharmacological compounds. In this study, PanaxGDB was constructed by using MySQL, ThinkPHP, and FastAdmin (http://panaxGDB.ynau.edu.cn; Figure 1). The database includes geographical distribution, morphology, and cultivar information on 16 *Panax* species. A section on the compounds of those species focuses on the distributions and characteristics of nearly 600 compounds as well as their metabolic pathways. For the published *P. notoginseng* genomes, the database also integrates the draft genomic sequence with structural and functional annotations and calculates the gene expression levels based on well-organized transcriptomes sampled from different tissues, growth stages, and treatments. The resequencing results of *P. notoginseng* were also uploaded on the Population page to perform genome-wide association analysis (GWAS) on agronomic traits associated with the yield of *P. notoginseng*. In addition, the powerful tools Blast and JBrowse are used to perform functional analysis.

## Materials and Methods

### Omics, Metabolome, and Germplasm Data Sets

The omics data set includes transcriptome sequencing, genomes, metagenomes, and chloroplast genomes. The bioinformation of the data sets was summarized, and a list was formed for easy searching. Germplasm data include species and cultivar information. Information was also collected on more than 500 compounds from 13 *Panax* species by searching the literature on phytochemistry between 1963 and 2021. The above information and that on chemical compositions were manually collected from publications, online resources, and other literature.

### Assembly and Gene Models of Genome Data

Genomic data sets primarily contain genome sequences, gene sequences, protein sequences, gff, gene functional annotations, and transcription factor (TF) annotations. Gene sequence and gff files of *P. ginseng* in the database were obtained from the ginseng genomic database (http://ginsengdb.snu.ac.kr/data.php). The 5th *P. notoginseng* genome assembly composed of 2.41 Gb from a previous study was included (Yang et al., 2021). Genome assemblies of *P. ginseng* and *P. notoginseng* included 59,352 and 47,870 genes, respectively. Genes were functionally annotated in InterPro (protein family; http://www.ebi.ac.uk/interpro/), pfam (protein family; http://pfam.xfam.org/), GO (Gene Ontology; http://geneontology.org/), NCBI non-redundant protein (Nr) database (http://www.ncbi.nlm.nih.gov), and Kyoto Encyclopedia of Genes and Genomes (KEGG) database (http://www.genome.jp/kegg) by using BLASTX (e-value < 10^−5^; http://www.ncbi.nlm.nih.gov/BLAST/). The transcription factors were identified with PlantTFDB serve (http://planttfdb.gao-lab.org/).

### Digital Gene Expression Profiles

RNA-Seq data sets of *P. ginseng* and *P. notoginseng* with biological duplication were selected. Data sets included different tissues, development times, and treatments. Filtered RNA-seq was mapped to the latest reference genome by HISAT2 (Zhang et al., 2021). Matched reads were subsequently presented to StringTie for *de novo* assembly, during which the expression level of each gene and the isoform was estimated (Pertea et al., 2015). Gene expression maps were prepared by manual selection on the Gene page.

### Resequencing of *Panax notoginseng*

FastQC software (v0.11.5) was used for quality control of raw reads from Illumina HiSeqTM2000 (WuHang, HuNan, China) sequencing, and clean reads were mapped to the latest reference genome using the BWA software (v0.7.15-R1140; Li and Durbin, 2009; De Sena Brandine and Smith, 2019). The GATK software (v4.0.9.0) was used for single nucleotide polymorphisms (SNPs) identification (Tajima, 1989). The ANNOVAR software was used to calculate the number of mutation types of SNP sites in each sample (Wang et al., 2010). The software LUMPY (v0.2.13) was used to detect structural variation (SV) in this project (Layer et al., 2014). PCA of the sample group structure was performed by using PLINK (v1.9b6) and EIGENSOFT (EIG-6.1.4) software (Patterson et al., 2006; Price et al., 2006; Purcell et al., 2007). The software ADMIXTURE (v1.3.0; Alexander et al., 2009) was used to analyze population structure. The software SNPhylo 2 (Lee et al., 2014) was used to construct evolutionary relations between different samples, and Phylogeny.IO (https://github.com/oist/phylogeny-io) was used to construct the phylogenetic tree. A graph of linkage disequilibrium was obtained by PopLDdecay (v3.40) analysis (Zhang et al., 2019). GCTA (v1.93.2) software was performed for GWAS analysis, and a mixed linear model (MLM) was adopted as a modeling method (Yang et al., 2011).

### Transcriptome of *Panax notoginseng*

A total of 20.3 G of PacBio Iso-Seq data and 92.6 G of raw RNA-seq data of three representative tissues (roots, stems, leaves) were acquired from *P. notoginseng* sequencing. The RNA from the three tissues was mixed to establish two SMRT cells for SMRT sequencing. Smrtlink 5.0 software was used to process the raw data, and the obtained redundant sequences were clustered by an iterative clustering for error correction algorithm to obtain full-length consensus sequences (FL; Pertea et al., 2015). Quiver was used to align FL sequences and non-FL reads. The high-quality sequence with accuracy >0.99 obtained in the previous step was further aligned with the assembled data from Illumina RNA-seq by using Lordec (v0.9) and mapped to the reference genome by using GMAP software (Wu and Watanabe, 2005).

### Database Implementation

PanaxGDB is based on the Apache web server (http://www.apache.org), adopted ThinkPHP5.1 (http://www.thinkphP.cn)-based FastAdmin templates, included frameworks of Codelgniter (https://www.codeigniter.com/) and Bootstrap (https://getbootstraP.com), and applied programming languages including CSS, PHP-HTML5, and JavaScript. MySQL (https://www.mysql.com) was used for data sorting, storage, and management, and the AJAX asynchronous loading scheme was used for quick data loading and function implementation. To provide an interactive user experience, Echarts (https://echarts.apache.org/zh/index.html), JBrowse (http://jbrowse.org), Phylogeny.IO (https://github.com/oist/phylogeny-io), and JQuery (https://jquery.com) were applied. The File Transfer Protocol-based download function was also provided with a transfer speed of up to 50 Mbps. Additionally, all functions in PanaxGDB were tested for web browsing on Safari, Chrome, Firefox, IE, and Edge by mobile phone, pad, and computer.

## Results

### Database Structure

PanaxGDB with a user-friendly web interface includes nine pages: Home, Species, Cultivars, Gene, Compounds, Population, Tools, and Download (Figure 1). The Home page presents an overview of PanaxGDB as well as links to popular ginseng sites and biological tools. Other pages represent the five main topics of PanaxGDB.

### Germplasm of *Panax*

**Figure 2:**
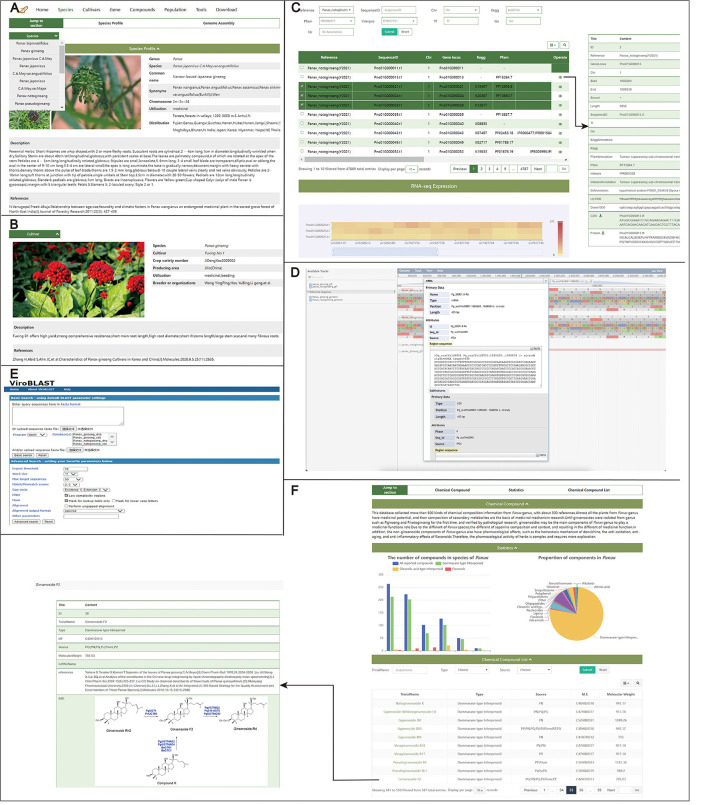
Query interface to retrieve information on germplasm, genomic application, and compounds. **(A)** Species page.16 species information on this page. **(B)** Cultivars page.62 cultivars information on this page. **(C)** Gene page. Search for genetic information including functional annotation, CDS, and protein sequence, etc. JBrowse **(D)** based on JavaScript for visual analysis of the genome annotation. Blast server **(E)** to perform homology searches with different data sets. **(F)** Compounds page. Search for the distribution, known metabolic pathways, or other characteristics of a particular compound.

Two modules of the germplasm resources of *Panax* are included in PanaxGDB: Species (Figure 2A) and Cultivars (Figure 2B). These two pages can help users quickly understand the species structure of *Panax* and the breeding status of *Panax* cultivars, respectively. To determine species, authoritative plant taxonomic keys were used. Species not authenticated were identified according to morphological and molecular data in various public references, and species of uncertain status were indicated. *Panax assamicus* found in northeast India, which was not accepted by the Board of Trustees of the Royal Botanic Gardens, was cited as *P. bipinnatifidus* var. *angustifolius*. Similarly, *Panax shangianus* and *Panax omeiensis* were not included in the authoritative source for plant nomenclature. Although *Panax vietnamensis* var. *fuscidiscus* and *P. vietnamensis* var. *langbianensis* were not included in authoritative plant taxonomies, morphological and molecular evidence indicate they are new varieties (Van Duy et al., 2016).

Sixteen *Panax* species are summarized in the database, and eight categories of information are provided for each species on the Species page, including pictures, common name, synonyms, chromosome number, utilization, distribution, description, and references (Figure 2A). In China, local provincial authorities register and identify cultivars of medicinal plants, and as result, information on various cultivars is not concentrated. Therefore, enterprises and producers involved in *Panax* cultivation are limited in the collection of cultivar information, which is difficult to meet market demand. On the Cultivars page, those participating in the Panax industry can easily obtain the current breeding status of Panax cultivars to screen optimal cultivars for different products under local environmental conditions. The Cultivars page contains eight categories of information on cultivars, including crop variety number, uses, area of production, description, pictures, breeders or organizations, and references (Figure 2B). A total of fifty *P. ginseng* cultivars are recorded in PanaxGDB, including 19 from China, 30 from Korea, and one from Japan, as well as two cultivars of *P. quinquefolius*, one of *P. japonicus*, and nine of *P. notoginseng* from China.

### Genetic Information

PanaxGDB provides viewing of the latest assemblies of *Panax* genomes (*P. ginseng* and *P. notoginseng*). Genome assembly files of *P. notoginseng* are open to the public for research for the first time. For convenience and practicality, genome assemblies were re-annotated and uploaded. On the Gene page, users can enter a TF, GO, and KEGG orthology identifiers (IDs), Pfam domain, InterPro domain, or gene ID in different search boxes to obtain detailed corresponding information, such as structural and functional annotation of genes of interest. For each gene, 22 categories of information are provided, such as gene locus, length, and downloadable sequence, among others, which can be displayed using the “Operate” option in the upper-right of the display list (Figure 2C). Users can also access the ginseng genome databases from the Home page. In the gene expression module, one or more genes can be selected according to specific needs, and gene expression levels of different groups or treatments can be visualized from a heat map of ticked, related genes. Heat maps provide information on data accession numbers and associated reference (Figure 2C).

On the Tool page, JBrowse and Blast can help users visualize *Panax* genomes and sequence alignments. Currently, *P. ginseng* and *P. notoginseng* genomes, gene annotations, and information on *Panax* variants can be imported into JBrowse. Sets of genomes, gene and density information on SNP/300 kb, and allele frequency can be explored in PanaxGDB in JBrowse (Figure 2D). The Blast tool is provided for sequence alignment with Sequence coding for amino acids in protein (CDS) and protein sequences of *P. ginseng* and *P. notoginseng* and uses the input of a similar sequence (Figure 2E). The final Blast result can be downloaded in HTML format.

### Metabolites of *Panax*

Secondary metabolites in *Panax* are pharmacologically active. Therefore, a metabolites page was provided with an interface (Compounds page) to retrieve, visualize, and investigate metabolite distribution in 12 *Panax* species (Figure 2F). The page includes 587 compounds of *Panax*, of which 492 are saponins. Damarane-type saponins accounted for 76.8% (451) of the known compounds. More than 200 saponins have been detected in *P. ginseng* and *P. notoginseng* according to the available phytochemical and pharmacological literature. In the Statistics modules, the proportion, quantity, and distribution of different types of compounds across different species can be visualized. In the list of chemical compounds, users can find ginsenoside Rb1, Rb2, Rc, Rd, Rb3, Re, Rg1, 20(S)-Rg2, Rh1, Rf, and Ro, notoginsenoside R1, and gypenoside XVII in at least eight *Panax* species. More than two *Panax* species contain 148 types of saponins. However, some characteristic saponins, such as quinquenoside I, II, III, IV, and V, are found only in *P. quinquefolium* (Yoshikawa et al., 1998). According to statistics, characteristic saponins are found in *P. ginseng, P. notoginseng, P. quinquefolius, P. japonicus, P. japonicus* var. *major, P. vietnamensis, P. stipuleanatus*, and *P. bipinnatifidus*. However, the list might be incomplete because of insufficient detection depth. To search for additional information, users can search by entering the name of a specific compound, compound type, or species origin. Users can click on a metabolite to link the metabolite information subpage and complete metabolite information, including biochemistry, biosynthesis pathway, and its species origin. Another hidden effect of this page is to help understand validated biosynthetic pathways of compounds, especially saponins, and validated genes in those pathways.

### Population Structure

**Figure 3:**
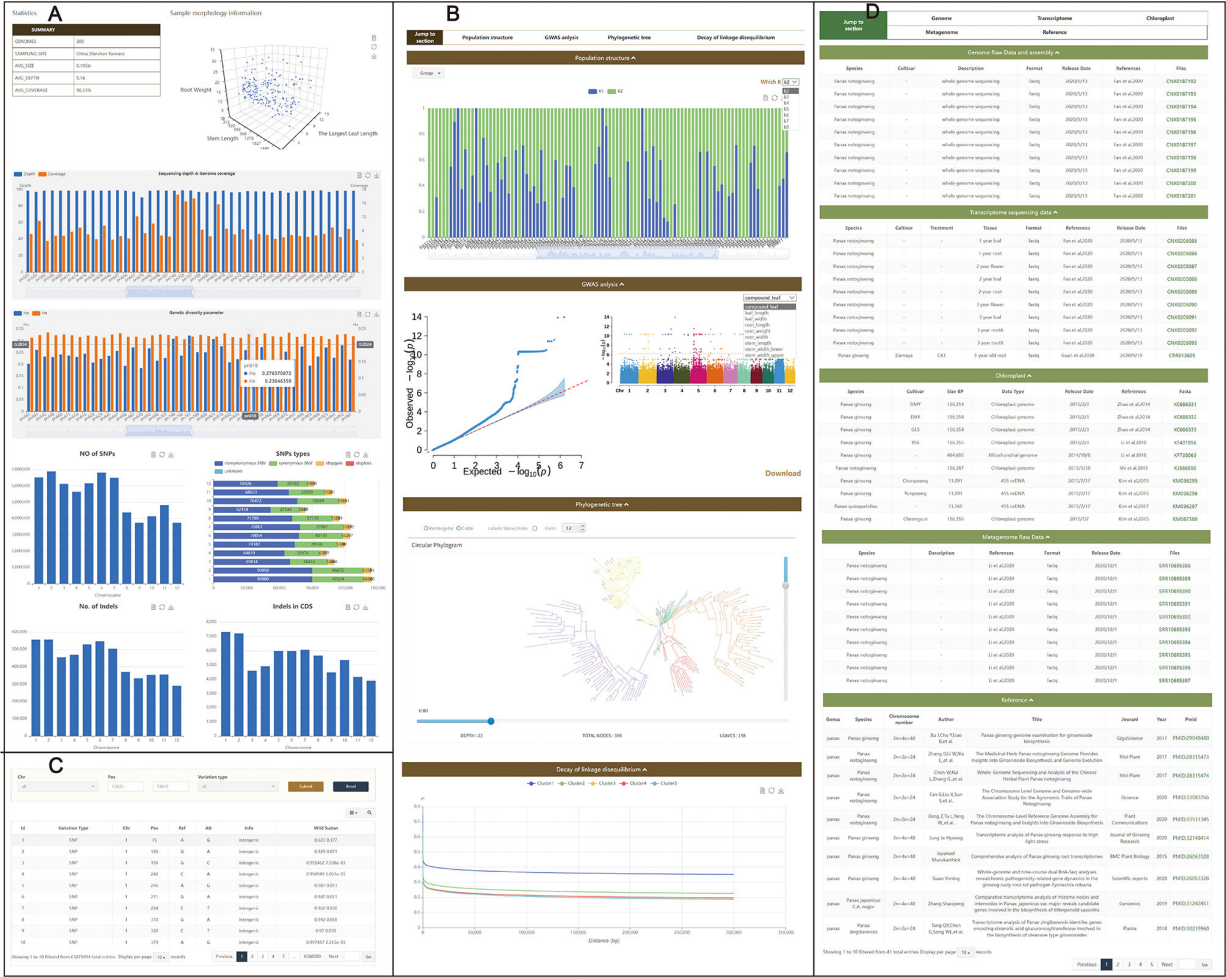
Page on population and download. Population page contains three subpages: Statistics **(A)**, Analysis **(B)**, and Variations **(C)**. Download page **(D)**, summary and presentation of Panax omics information including transcriptome sequencing, genome, metagenome, and chloroplast genome.

The resequencing database was set for *Panax* breeding and germplasm resource management. Data from *P. notoginseng* were collected on the Population page, and 200 3-year-old *P. notoginseng* samples from Yunnan, China, with nine important agricultural traits were randomly selected for resequencing. The average sequencing size of each sample was 28.83 Gb. Results were visualized and uploaded to the Population page with three modules which included Statistics (Figure 3A), Analysis (Figure 3B), and Variations (Figure 3C). Statistical results of the resequencing can be seen on the Statistics subpage. The Analysis subpage includes population structure, phylogenetic tree, decay of linkage disequilibrium, and Manhattan figure. All images and corresponding data on this page can be downloaded. Users can also search for SNPs on the Variations subpage based on results of phenotype evaluation and GWAS.

A total of 1,615,492,994 SNPs were obtained in 200 samples. There were 1,462,055,399 SNPs of 200 samples within intergenic regions, 114,939,464 within intronic regions, and 38,498,131 within exons. These data can be visualized on the Statistics subpage. On the Analysis subpage, the phylogenetic tree was derived from the SNP data, and the tree shows *P. notoginseng* of Wenshan and can be divided into five subgroups. In the GWAS module, there were 192 highly significant peaks on 12 pseudochromosomes associated with traits (root weight and leaf width) related to saponin yield. Those SNPs can be found on the Variations pages, and 22 occurred in CDSs (Supplementary Table 1), which can be found on the Gene page. Those genes were annotated as proteins associated with cell metabolism and growth, including peptidyl-prolyl cis-trans isomerase, aminotransferase, oxoglutarate dioxygenase, 2-isopropylmalate synthase, WD40-repeat protein, AMP-dependent synthetase, and phosphatidylinositol N-acetylglucosaminyltransferase, protein kinases, or as plant stress-related proteins, including heat shock protein (Supplementary Table 1).

### Omics Data Acquisition and Upload

To make it more convenient to use *Panax* omics data, all publicly available data were gathered and classified. The Download page contains information and links to all available public *Panax* omics data, and long-read sequencing and resequencing data were published for the first time in PanaxGDB (Figure 3D). Downloadable content in this module includes 405 transcriptome sequencings, two genomes, 21 metagenomes, and 67 chloroplast genomes. The visual data can help users to summarize information. Because the database is an open platform, prospective ginseng omics data are welcomed and can be included in the database by contacting the authors through the Data Submission page.

## Discussion

An early ginseng genome database was limited to the genome and transcriptome of *P. ginseng*, and it was not possible to visually compare expression levels of more than two genes (Jayakodi et al., 2018). In PanaxGDB, additional transcriptome data from different tissues and experiments were re-assembled, and information on those data, including sources and references, can be intuitively visualized, including comparisons of expression levels of different genes. PanaxGDB provides the most comprehensive overview of the omics, metabolome, and germplasm resources of *Panax* and forms a user-friendly interface. In addition, the most complete analysis of resequencing and annotation results of the 5th version of the *P. notoginseng* genome and the full-length RNA-seq of *P. notoginseng*, as well as *Panax* cultivars, are fully displayed.

PanaxGDB is introduced as a comprehensive database with the first highly integrated multidirectional research results of the *Panax* genus to improve the practical use of data. PanaxGDB can be an important resource for those working with *Panax* in comparative studies of major cultivars, in the development of pharmacological value, and in heterogeneous biosynthetic research on saponins. As an open platform, advanced users are welcome to submit data related to *Panax* species to regularly update the database. Thus, PanaxGDB can continuously serve as a central portal to boost research on the biology and chemistry of *Panax*.

## Data Availability Statement

All data of this study are available through the PanaxGDB database website (http://panaxGDB.ynau.edu.cn).

## Author Contributions

XuL and SY: conception and design of the study. YLin, YLi, ZY, GX, YLu, QZ, XueL, GL, and BH: acquisition of data or analysis and interpretation of data. YLin, XuL, YD, GZ, and QY: database construction and visualization. YLin, BH, XuL, and SY: drafting of the article or revising it critically for important intellectual content. YLin, BH, YLu, YLi, YD, ZY, GX, GL, XueL, QZ, QY, GZ, SY, and XuL: final approval of the version to be published.

## Funding

This work was supported by National Key R & D Plan (2017YFC1702500), Major Science and Technology Projects in Yunnan Province (2019ZF011-1), Science and Technology Innovation team of Yunnan (202105AE160011), the First Projects of Science and Technology Plan in the Biomedical field in 2021 (202102AA310048), and the Digitalization of Biological Resource Project (202002AA1000051), Guangxi Innovation Driven Development Project (GuiKe AA18242040).

## Conflict of Interest

The authors declare that the research was conducted in the absence of any commercial or financial relationships that could be construed as a potential conflict of interest.

## Publisher's Note

All claims expressed in this article are solely those of the authors and do not necessarily represent those of their affiliated organizations, or those of the publisher, the editors and the reviewers. Any product that may be evaluated in this article, or claim that may be made by its manufacturer, is not guaranteed or endorsed by the publisher.
